# Author Correction: Comparative and demographic analysis of orang-utan genomes

**DOI:** 10.1038/s41586-022-04799-7

**Published:** 2022-08-12

**Authors:** Devin P. Locke, LaDeana W. Hillier, Wesley C. Warren, Kim C. Worley, Lynne V. Nazareth, Donna M. Muzny, Shiaw-Pyng Yang, Zhengyuan Wang, Asif T. Chinwalla, Pat Minx, Makedonka Mitreva, Lisa Cook, Kim D. Delehaunty, Catrina Fronick, Heather Schmidt, Lucinda A. Fulton, Robert S. Fulton, Joanne O. Nelson, Vincent Magrini, Craig Pohl, Tina A. Graves, Chris Markovic, Andy Cree, Huyen H. Dinh, Jennifer Hume, Christie L. Kovar, Gerald R. Fowler, Gerton Lunter, Stephen Meader, Andreas Heger, Chris P. Ponting, Tomas Marques-Bonet, Can Alkan, Lin Chen, Ze Cheng, Jeffrey M. Kidd, Evan E. Eichler, Simon White, Stephen Searle, Albert J. Vilella, Yuan Chen, Paul Flicek, Jian Ma, Brian Raney, Bernard Suh, Richard Burhans, Javier Herrero, David Haussler, Rui Faria, Olga Fernando, Fleur Darré, Domènec Farré, Elodie Gazave, Meritxell Oliva, Arcadi Navarro, Roberta Roberto, Oronzo Capozzi, Nicoletta Archidiacono, Giuliano Della Valle, Stefania Purgato, Mariano Rocchi, Miriam K. Konkel, Jerilyn A. Walker, Brygg Ullmer, Mark A. Batzer, Arian F. A. Smit, Robert Hubley, Claudio Casola, Daniel R. Schrider, Matthew W. Hahn, Victor Quesada, Xose S. Puente, Gonzalo R. Ordoñez, Carlos López-Otín, Tomas Vinar, Brona Brejova, Aakrosh Ratan, Robert S. Harris, Webb Miller, Carolin Kosiol, Heather A. Lawson, Vikas Taliwal, André L. Martins, Adam Siepel, Arindam RoyChoudhury, Xin Ma, Jeremiah Degenhardt, Carlos D. Bustamante, Ryan N. Gutenkunst, Thomas Mailund, Julien Y. Dutheil, Asger Hobolth, Mikkel H. Schierup, Oliver A. Ryder, Yuko Yoshinaga, Pieter J. de Jong, George M. Weinstock, Jeffrey Rogers, Elaine R. Mardis, Richard A. Gibbs, Richard K. Wilson

**Affiliations:** 1 The Genome Center at Washington University, Washington University School of Medicine, Saint Louis, Missouri USA; 2 Human Genome Sequencing Center, Department of Molecular and Human Genetics, Baylor College of Medicine, One Baylor Plaza, Houston, Texas USA; 3 MRC Functional Genomics Unit and Department of Physiology, Anatomy and Genetics, University of Oxford, Le Gros Clark Building, Oxford, UK; 4 Wellcome Trust Centre for Human Genetics, Oxford, UK; 5 Department of Genome Sciences, University of Washington School of Medicine, Seattle, Washington USA; 6 IBE, Institut de Biologia Evolutiva (UPF-CSIC), Universitat Pompeu Fabra, PRBB, Doctor Aiguader, 88, Barcelona, Spain; 7 Howard Hughes Medical Institute, Seattle, Washington USA; 8 Wellcome Trust Sanger Institute, Wellcome Trust Genome Campus, Cambridge, UK; 9 European Bioinformatics Institute, Wellcome Trust Genome Campus, Hinxton, Cambridge UK; 10 Center for Biomolecular Science and Engineering, University of California, Santa Cruz, California USA; 11 Center for Comparative Genomics and Bioinformatics, Penn State University, University Park, Pennsylvania, USA; 12 CIBIO, Centro de Investigação em Biodiversidade e Recursos Genéticos, Universidade do Porto, Campus Agrário de Vairão, Vairão, Portugal; 13 Instituto de Tecnologia Química e Biológica, Universidade Nova de Lisboa, Oeiras, Portugal; 14 ICREA (Institució Catalana de Recerca i Estudis Avançats) and INB (Instituto Nacional de Bioinformática) PRBB, Doctor Aiguader, 88, Barcelona, Spain; 15 Department of Biology, University of Bari, Bari, Italy; 16 Department of Biology, University of Bologna, Bologna, Italy; 17 Department of Biological Sciences, Louisiana State University, Baton Rouge, Louisiana USA; 18 Center for Computation and Technology, Department of Computer Sciences, Louisiana State University, Baton Rouge, Louisiana USA; 19 Institute for Systems Biology, Seattle, Washington USA; 20 Department of Biology and School of Informatics and Computing, Indiana University, Bloomington, Indiana USA; 21 Instituto Universitario de Oncologia, Departamento de Bioquimica y Biologia Molecular, Universidad de Oviedo, Oviedo, Spain; 22 Faculty of Mathematics, Physics and Informatics, Comenius University, Mlynska Dolina, Bratislava, Slovakia; 23 Institut für Populations genetik, Vetmeduni Vienna, Wien, Austria; 24 Department of Anatomy and Neurobiology, Washington University School of Medicine, Saint Louis, Missouri USA; 25 Department of Biological Statistics and Computational Biology, Cornell University, Ithaca, New York USA; 26 Department of Biostatistics, Columbia University, New York, New York USA; 27 Department of Genetics, Stanford University, Stanford, California USA; 28 Department of Molecular and Cellular Biology, University of Arizona, Tucson, Arizona USA; 29 Bioinformatics Research Centre, Aarhus University, Aarhus C, Denmark; 30 San Diego Zoo’s Institute for Conservation Research, Escondido, California USA; 31 Children’s Hospital Oakland Research Institute, Oakland, California USA; 32 Present Address: Department of Bioengineering, University of Illinois at Urbana-Champaign, Urbana, Illinois USA

Correction to: *Nature* 10.1038/nature09687 Published online 26 January 2011

In this Article, we reported genome sequencing data from five Sumatran and five Bornean orang-utans. In re-analysing these data, Banes et al.^1^ found that eight of the ten samples were mislabelled, and we wish to correct the original publication according to their findings. In addition, Banes et al.^1^ find that one of the orang-utans that we had labelled as a male Sumatran is in fact a female Tapanuli orang-utan (an orang-utan species that was unknown at the time of publication of our original Article and that was first reported in 2017 (ref. ^2^)). While we here correct the original tables to display the correct names and IDs of the individuals studied, we do not correct the analyses in the original paper since the Tapanuli species was discovered only after we had concluded our study. We refer the reader to Banes et al.^1^ for further information.

In the main text, the sentence “We also sequenced the genomes of 10 additional unrelated wild-caught orang-utans, five Sumatran and five Bornean, using a short read sequencing platform (297 Gb of data in total; Supplementary Information section 4)” should have read “We also sequenced the genomes of 10 additional unrelated wild-caught orang-utans, **four** Sumatran**, five** Bornean **and one Tapanuli**, using a short read sequencing platform (297 Gb of data in total; Supplementary Information section 4)”.

In addition, the sentence “We then called single nucleotide polymorphisms (SNPs) from the alignment of all short read data from 10 individuals (five Bornean, including the 20-fold coverage mentioned above, and five Sumatran) (Supplementary Information section 4)” should have read “We then called single nucleotide polymorphisms (SNPs) from the alignment of all short read data from 10 individuals (five Bornean, including the 20-fold coverage mentioned above, **four Sumatran and one Tapanuli**) (Supplementary Information section 4).

Supplementary Data to this Amendment corrects the sample ID in the original Supplementary Data (‘Accession Table for Bornean and Sumatran Orangutan Short Read Data’) for animal sample ID KB9258 from a Sumatran male to a Tapanuli female (line 204). In Supplemental Section S4 of the original Supplementary Information, the first sentence “The orangutan population diversity survey utilized DNA from 5 Sumatran and 5 Bornean wild-caught orang-utans […]” should have read “The orangutan population diversity survey utilized DNA from **4 Sumatran, 5 Bornean and 1 Tapanuli** wild-caught orang-utans […]”. Supplementary Table 1 to this Amendment shows a corrected version of the original Supplementary Table S4-1, reproduced with modifications from Banes et al.^1^.

None of these corrections affect the results and conclusions of the original Article with the exception of Fig. 5 in the main text. The estimates of SNPs in panel **a**, allele counts in panel **b** and estimates of population (*N*_e_) should be recalculated for the Sumatran and Tapanuli separately in the future. Our Bornean estimates in Fig. 5 are unaffected by this sample mislabelling, as noted in Banes et al.^1^. The original Article has not been corrected online.

## Supplementary information

Supplementary Table 1 A corrected version of the original Supplementary Table S4-1 (Next generation sequence data summary).

Supplementary Data A corrected version of the original Supplementary Data (Accession table for Bornean and Sumatran orang-utan short read data).

## References

[1] Banes, G. et al. Nine out of ten samples were mistakenly switched by The Orang-utan Genome Consortium. *Sci. Data* (in the press). 10.1038/s41597-022-01602-0 PMC9374732 35961988

[2] Nater A (2017). Morphometric, behavioral, and genomic evidence for a new orangutan species. Curr. Biol..

