# Supplementary material for: Author Correction: Comparative and demographic analysis of orang-utan genomes
Source: Nature. 2022 Aug 12;608(7924):E36. doi: 10.1038/s41586-022-04799-7 (PMC9402433; doi:10.1038/s41586-022-04799-7)
Supplement: Supplementary file 1 — A corrected version of the original Supplementary Table S4-1 (Next generation sequence data summary). [file 41586_2022_4799_MOESM1_ESM.docx]

**Table S4-1. Next generation sequence data summary**

| **Sample ID** | **Studbook#** | **Name** | **Institution When Sampled** | **Origin** | **Sex** | **Raw Data** | **Coverage (x)** |
| --- | --- | --- | --- | --- | --- | --- | --- |
| KB5404 | 356 | Dinah | Dallas zoo | Bornean | F | 61 | 20.3 |
| KB5406 | 364 | Dolly | Dallas zoo | Bornean | F | 23 | 7.7 |
| KB5405 | 360 | Dennis | Dallas zoo | Bornean | M | 26 | 8.7 |
| KB4204 | 590 | Billy | Lincoln Park Zoo, Chicago | Bornean | M | 25 | 8.3 |
| KB5543 | 990 | Louis | Los Angeles Zoo | Bornean | M | 29 | 9.7 |
| SB550 | 550 | Sibu | Altanta Zoo | Sumatran | F | 21 | 7 |
| KB9528 | 695 | Bubbles | San Diego Zoo | Tapanuli | F | 28 | 9.3 |
| KB4361 | 53 | Doris | San Diego Zoo | Sumatran | F | 21 | 7 |
| KB4661 | 732 | Baldy | Sacremento Zoo | Sumatran | M | 20 | 6.7 |
| KB5883 | 1600 | Likoe | Miami Metro Zoo | Sumatran | M | 25 | 8.3 |
